# Moral Lacunae in the Management of Dual Agency Dilemmas: Comment on "Dual Agency in Hospitals: What Strategies Do Managers and Physicians Apply to Reconcile Dilemmas Between Clinical and Economic Considerations?"

**DOI:** 10.34172/ijhpm.2022.6779

**Published:** 2022-03-10

**Authors:** Wendy Lipworth

**Affiliations:** Department of Philosophy, Macquarie University, Sydney, NSW, Australia.

**Keywords:** Dual agency, Resource Allocation, Conflict of Interest

## Abstract

Waitzberg and colleagues’ participants articulate a wide range of strategies to manage tensions between clinical and economic obligations. There are, however, three notable absences in the data. First, all strategies described by participants are underpinned by the assumption that clinical (and associated administrative) practices *need* to either align with economic considerations or be made more compatible with them. Second, the dual agency dilemma was framed exclusively as existing at the level of the health care institution, with little attention paid to obligations to broader health systems. Third, there was no evidence of critical questioning of the priorities of the hospitals in which participants work. These absences do not render the strategies used by Weitzberg and colleagues’ participants morally "wrong," but they do suggest that people who are deeply embedded in a system might fail to recognise the full range of moral concerns and moral possibilities.

 Waitzberg et al^1^ provide a compelling account of the strategies used by health care providers and managers who are “dual agents” and who need to balance clinical and economic considerations in their work. Some of these strategies have been identified by others see, for example.^2-4^ What Waitzberg and colleagues’ analysis adds is insight into how a particular kind of (activity-based) payment system creates particular kinds of dual agency dilemmas and motivates particular kinds of management strategies.

 The data produced by the study is rich, with participants describing a wide range of strategies aimed at managing the tensions they face. There are, however, three interesting and potentially morally significant “absences” in Waitzberg and colleagues’ findings.

 The first absence relates to the ways in which participants reflected on possible “ways out” of their dilemma. They described how activity-based payment might incentivize proper treatment, and how clinical and economic considerations might be aligned by improving efficiency or specialisation. They also described a range of ways of managing competing obligations when such alignment is not possible, including reshaping managerial practices and reframing the focus of decision-making to bigger units of analysis.

 While these are all distinct strategies, they are all underpinned by the assumption that clinical (and associated administrative) practices *need *to either align with economic considerations or be made more compatible with them. There is, however, one other way in which the dilemma between clinical and economic considerations could be addressed, and that is by physicians determining that they are responsible solely for patient care and leaving it to managers to address economic considerations.

 This strategy, which has been referred to as “bunkering,”^5^ is justified primarily on the basis that physicians have one clear primary obligation that outweighs all others: to do whatever is in the best interests of the patient in front of them. Bunkering has also been justified on the basis that it is not necessary for doctors to pay attention to economic considerations because there are other, more significant, causes of inefficiency than doctors advocating solely for their patients. Other arguments against doctors simultaneously considering clinical and economic considerations are that doing so is not workable (because there are no “rational” ways of doing it), not procedurally just (because some patients lose out more than others), not ultimately effective for reallocation of resources, and not compatible with trust in health care professionals.^5^

 There is evidence from other empirical research that some health professionals choose “bunkering” as their strategy for managing the tensions between clinical and economic imperatives. In an interview study exploring Australian physicians’ prescribing of high-cost cancer medicines,^6,7^ for example, some participants argued that (unpublished data):

*“Individual doctors cannot provide the checkpoints….as doctors we need to be advocating the best we can for the patient who is in front of you.” *

 To argue for the primacy of the patient is not to say that health professionals should have no concern for other goods, but rather that they should be clear about what “hat” they are wearing at any given time. For example, a doctor might advocate solely for his or her patients when in a clinical setting, but also sit on a hospital resource allocation or formulary committee, or at least participate in gathering data to support such processes. The decisions made by these committees might constrain subsequent practice (by, for example, excluding particular medicines from a hospital formulary) but the doctor is free to offer whatever is best for his or her patients within these constraints.

 This approach (which is sometimes referred to as “role morality”) is not without its challenges—for example physicians participating in macroallocation processes might have difficulty setting aside their desire to advocate for their own patients, or have concerns about the justice of the processes in which they are participating.^8^ But the advantage of role morality over strict bunkering is that a degree of social solidarity is maintained, while also resolving the clinician’s dilemma at the bedside.

 Waitzberg and colleagues’ participants alluded to role morality in their descriptions of planning ahead, developing tools for decision-making and making use of multidisciplinary decision-making. But even here, the purpose was not explicitly so that professionals could assume a singular, clinically focused, role at other times. In other words, there was no direct reference to role morality in these participants’ accounts.

 The absence in Waitzberg and colleagues’ study of bunkering and role morality could have been because the study was premised on the assumption that professionals working in hospitals experience dilemmas and have to manage them on a day-to-day basis. This could have affected the ways in which questions were constructed and obscured other strategies for managing dual agency. But given the prominence of these strategies in ethical debates about the management of dual agency, it is somewhat surprising that they did not emerge (even if only to be rejected).

 Another noteworthy absence in Weitzberg and colleagues’ results is that little attention is paid, either in the framing of the article or by those interviewed, to the obligations that doctors (and, indeed hospitals) might have to the broader health systems in which they are embedded. In this regard, it is noteworthy that several of the strategies described by participants, such as transferring patients to other parts of the health system or adjusting coding to improve reimbursement, could have adverse impacts on the broader health system. While it was acknowledged that these strategies could impact negatively o*n patients*, their effects on the health system as a whole did not seem to be a significant consideration.

 In contrast, in the study of cancer physicians in Australia, participants were acutely aware of their responsibilities to the health system (unpublished data):

*“We’ve got a responsibility to the broader community to spend the health dollar wisely.” *

 They saw themselves as being simultaneously clinicians and citizens (and taxpayers), and this is where their dilemmas lay;

 “*I have a view as a hopefully educated citizen and a view as a clinician, and they’re not necessarily concordant, because of the conflicting priorities that you have*.*”*

 Indeed, even those physicians in this study who advocated for “bunkering” did so in the knowledge that there would be society-based constraints on what they do, which would protect the broader health system:

 “*Individual doctors cannot provide the checkpoints….as doctors we need to be advocating the best we can for the patient who is in front of you…But overarching that there are some regulations you have to work within for the long term sustainability of Australia, and they are provided by the government*.*”*

 It might be the case that such concerns are unique to countries such as Australia, which have strong and highly valued public health care systems. But even in highly privatised systems, such as the United States, physicians are seen to have obligations to the health system as a whole. For example the American Board of Internal Medicine Foundation’s “Medical Professionalism in the New Millenium: A Physician Charter” emphasises not only the “primacy of the patient” (noting that “market forces, societal pressures, and administrative exigencies must not compromise this principle”) but also the need for social justice (noting that “[t]he medical profession must promote justice in the health care system, including in the fair distribution of resources”).^9^

 The absence of concern for health systems in Weitzberg and colleagues’ article might be a result of the scope of the study and the ways in which questions were framed—however it is noteworthy that in the Australian study, societal issues were raised frequently and spontaneously, even when questions were not explicitly asked about them, for example:

*“[I]t’s … my responsibility to be pushing back … as a taxpayer I feel very strongly that we have a responsibility to be advocating for the appropriate use of these innovative agents, particularly when they come at such a high cost...*”

 The fact that these issues were not raised by Weitzberg and colleagues’ participants could, therefore reflect a de-privileging of values such as solidarity—understood here as the act of publicly standing up to protect other patients from inequitable lack of access,^10,11^ which might be a function of working in largely privatised health systems. This is not to say that society should necessarily take precedence over individual patients (or hospitals), even though doing so might satisfy some accounts of justice,^12^ but it does remind us that there are other values that need to at least be considered when thinking through dual agency dilemmas.

 A third (and related) absence in Weitzberg and colleagues’ results is that none of the participants interviewed questioned the values, motives or priorities of their institutions and, correspondingly, their obligations to these institutions While it is true that health professionals need their organisations to survive and thrive, there is always room to question the policies and procedures that drive these institutions. No such critical questioning was evident in the strategies that Weitzberg and colleagues’ participants articulated. Of course, critical questioning on the part of health professionals would not necessarily lead to organisational or system change, but it is interesting that participants seemed to be uncritical of the types of organisations in which they are embedded.

 These three absences do not render the strategies used by Weitzberg and colleagues’ participants morally “wrong,” but they do show how efforts to do the right thing in the context of particular modes of reimbursement (in this case activity-based funding) might blind people to other moral concerns and other moral possibilities. Rather than simply accepting the status quo, and finding ways to work morally within it, it is important to consider more radical options that do not assume that existing models of funding need to be taken at face value or prioritised over either individual patients or broader health systems.

## Ethical issues

 Not applicable.

## Competing interests

 Author declares that she has no competing interests.

## Author’s contribution

 WL is the single author of the paper.

## Funding

 This work was supported by the National Health and Medical Research Council [APP APP1080673].
